# Logistic, ethical, and political dimensions of stepped wedge trials: critical review and case studies

**DOI:** 10.1186/s13063-015-0837-4

**Published:** 2015-08-17

**Authors:** Audrey Prost, Ariella Binik, Ibrahim Abubakar, Anjana Roy, Manuela De Allegri, Christelle Mouchoux, Tobias Dreischulte, Helen Ayles, James J. Lewis, David Osrin

**Affiliations:** Institute for Global Health, University College London, 30 Guilford Street, London, WC1N 1EH UK; The Ethox Centre, University of Oxford, Oxford, UK; Public Health England (PHE), Colindale, UK; Institute of Public Health, Faculty of Medicine, University of Heidelberg, Heidelberg, Germany; Hôpital des Charpennes, Hospices Civils de Lyon, Villeurbanne, France; Tayside Medicines Unit, NHS Tayside, Dundee, UK; MRC Tropical Epidemiology Group, Department of Infectious Disease Epidemiology, London School of Hygiene and Tropical Medicine, London, UK

**Keywords:** Stepped wedge trials, Ethics, Methodology, Public health

## Abstract

**Background:**

Three arguments are usually invoked in favour of stepped wedge cluster randomised controlled trials: the logistic convenience of implementing an intervention in phases, the ethical benefit of providing the intervention to all clusters, and the potential to enhance the social acceptability of cluster randomised controlled trials. Are these alleged benefits real? We explored the logistic, ethical, and political dimensions of stepped wedge trials using case studies of six recent evaluations.

**Methods:**

We identified completed or ongoing stepped wedge evaluations using two systematic reviews. We then purposively selected six with a focus on public health in high, middle, and low-income settings. We interviewed their authors about the logistic, ethical, and social issues faced by their teams. Two authors reviewed interview transcripts, identified emerging issues through qualitative thematic analysis, reflected upon them in the context of the literature, and invited all participants to co-author the manuscript.

**Results:**

Our analysis raises three main points. First, the phased implementation of interventions can alleviate problems linked to simultaneous roll-out, but also brings new challenges. Issues to consider include the feasibility of organising intervention activities according to a randomised sequence, estimating time lags in implementation and effects, and accommodating policy changes during the trial period. Second, stepped wedge trials, like parallel cluster trials, require equipoise: without it, randomising participants to a control condition, even for a short time, remains problematic. In stepped wedge trials, equipoise is likely to lie in the degree of effect, effectiveness in a specific operational milieu, and the balance of benefit and harm, including the social value of better evaluation. Third, the strongest arguments for a stepped wedge design are logistic and political rather than ethical. The design is advantageous when simultaneous roll-out is impractical and when it increases the acceptability of using counterfactuals.

**Conclusions:**

The logistic convenience of phased implementation is context-dependent, and may be vitiated by the additional requirements of phasing. The potential for stepped wedge trials to enhance the social acceptability of cluster randomised trials is real, but their ethical legitimacy still rests on demonstrating equipoise and its configuration for each research question and setting.

## Background

Stepped wedge trials are a type of cluster randomised controlled trial in which clusters are randomised to receive an intervention at different start times, and all clusters eventually receive it. Arguments for stepped wedge trials (henceforth referred to as SWT) fall into three broad categories. The first relates to logistic benefits: phased implementation of an intervention is advantageous when it is infeasible or impractical to introduce it in a large number of clusters simultaneously [4, 9, 27, 29, 46]. The second argument relates to ethical benefits. The fact that all clusters eventually receive the intervention is thought to alleviate concerns about denying benefits to control groups, especially when evidence suggests that the intervention is likely to have a positive effect compared to current practice [9]. A third, related, argument is that introducing an intervention to all clusters over time may make cluster randomised controlled trials (CRT) more socially acceptable, particularly in the context of implementation research nested within ongoing health programmes, or when further evidence is sought about an already accepted policy [52].

Despite these perceived benefits, SWT face criticisms. Some researchers argue that they are particularly susceptible to bias introduced through secular changes in main outcomes and usually take longer than parallel group trials to achieve equivalent statistical power [29, 39]. Others contend that they pose a greater risk of attrition than parallel group trials, and that it is difficult to justify the ethics and costs of delivering an intervention to all clusters if it is proven ineffective in the final analysis [39].

Previous discussions of the benefits and drawbacks of the stepped wedge design have tended to focus mainly on its statistical efficiency and analytical requirements. With notable exceptions, including the work of Kotz *et al*. [39], assumptions about its logistic, ethical, and political dimensions have generally been examined in theoretical rather than pragmatic terms. Are the alleged logistic, ethical, and political benefits of stepped wedge trials real or assumed? In this article, we explore the reality of implementing such trials, their ethical and political dimensions. We used data from the review conducted from this series [4], and examined six case studies of recent evaluations [14, 17, 35, 50, 58, 63].

## Methods

This article is part of a broader series on stepped wedge trials. We examined reasons for conducting SWT reported in studies published between 2010 and 2014 [4, 9, 46]. We then reviewed completed or ongoing trials to identify potential case studies, using the review published by Mdege *et al*., which included SWT published up till 2010, and the review by Beard *et al*. included in this collection [4, 46]. We did not use predefined inclusion or exclusion criteria to select case studies. Instead, the studies were purposively chosen to span a range of public health interventions and to include high-income and low-income settings. Drawing upon case-study approaches, two authors (AP and DO) interviewed the study authors and discussed their rationale for choosing a stepped wedge design, logistic issues faced during implementation, and ethical benefits and drawbacks [12]. We reviewed the interview transcripts, identified emerging issues through qualitative thematic analysis, reflected upon them in the context of the literature, and invited all participants to comment on the manuscript as co-authors [59, 60]. We obtained informed consent from all participants. As participants were considered to be ‘key informants’ and participated in reviewing drafts of the article as co-authors, no specific ethical approval was required or obtained for this work.

## Results

Table 1 summarises the characteristics of the stepped wedge evaluations in which six co-authors participated (IA, AR, MDA, CM, TD, HA). Two were conducted in the UK, one in France, two in Zambia, and one in Burkina Faso. Three studies tested complex interventions to improve the quality of care in health facilities: the Data-driven Quality Improvement in Primary Care (DQIP) study in general practice surgeries in the UK, the CONFUCIUS study in French surgical wards, and the BHOMA study in Zambian primary health centres [17, 50, 63]. One study tested the effect of introducing routine HIV testing at tuberculosis clinics in London, UK [35, 58]. Another assessed the effect of community health insurance on access to care and household spending in Burkina Faso [14]. Finally, a non-randomised stepped wedge evaluation compared two ways of delivering antiretroviral therapy for HIV to pregnant women in Zambia [35].

**Table 1:** Characteristics of trials led by the authors

Lead author	Country	Clusters	Intervention	Main outcome(s)	Interview number
De Allegri [14]	Burkina Faso	33 geographical rural and urban clusters	• Offer of community health insurance	Health service utilization	1
Dreischulte [17] (DQIP study)	United Kingdom	40 general practice surgeries in two Scottish health boards	• Each practice received a visit providing education on targeted prescribing and training in the use of an informatics tool	Composite measure of high-risk prescribing	2
			• The informatics tool provided weekly updated feedback of targeted prescribing at practice level, prompting the review of individual clients affected, and summarising each patient’s relevant risk factors and prescriptions		
			• Payments for practices: a US $560 upfront incentive and US $24 for each client reviewed during the intervention		
Killam [35]	Zambia	Antenatal care clinics	• Antiretroviral therapy for HIV during antenatal care	Proportion of treatment-eligible women initiating antiretroviral therapy during pregnancy	3
Roy [58]	United Kingdom	Tuberculosis clinics in London	• Universal offer of HIV testing in tuberculosis clinics	Levels of HIV test offers, acceptance and coverage	4
Mouchoux [50] (CONFUCIUS study)	France	Three surgical wards	• Preoperative geriatric consultation	Postoperative delirium within seven days of surgery	5
			• Training of ward staff and Hospital Elder Life Program		
			• Conferences about cases of delirium		
Stringer [63] (BHOMA study)	Zambia	Catchment areas of primary health care centres	• Implementation of clinical protocols, forms, and systems by Quality Improvement (QI) team in primary health centres.	Overall mortality	6
				Under-five mortality	
			• Monitoring of care and mentoring facility staff to improve quality.		
			• Engagement of community health workers to refer and follow up clients.		

### Logistic features

#### Phased implementation: useful but not necessarily easy

‘My own personal perspective is that this is quite a seductive design, because of the practical aspects, the fact that everybody gets the intervention, the fact that you can look at the effect of time on the impact of the intervention. The big caveat is that it requires ‘extreme coordination’ to achieve all the different tasks. In each step, you have to ensure that each clinical service has included enough patients, because otherwise you miss out… So you have very rigorous parameters to abide by. And that, for me, is the only drawback’. (Interview 5)

‘As soon as you randomise the practices, you need to basically get a date in your diary when you initiate the intervention. And we were struggling at the beginning - because the practices were busy’. (Interview 2)

In 20 of the 37 SWT reviewed for this series, and in all six case studies, the possibility of implementing the intervention in phases was either the main or an important reason for choosing the design [2, 5, 6, 8, 13, 17, 19, 23, 36, 37, 43, 50, 54, 56, 61]. In Burkina Faso, the community health insurance scheme being tested could not logistically have been implemented in all clusters at once; neither could the quality-improvement intervention implemented in one of the two Zambian trials [14, 63]. In the non-randomised stepped wedge evaluation of antiretroviral provision in Zambian antenatal clinics, phased implementation was desirable because there were substantial differences in clinic size and patient numbers, and the intervention team wanted to begin with smaller clinics before tackling larger ones [35]. Logistics also have ethical implications: if it is unfeasible or very challenging for an organisation to roll out an intervention throughout an area or health service, it is likely that a better intervention will be delivered by phasing its implementation.

However, phasing the intervention and adhering to randomised implementation schedules often introduced new challenges. In the case of complex interventions with multiple components, it typically meant scheduling several rounds of training activities. Five of our six case studies took place within health systems, and phased implementation was challenging when communicating with busy clinical teams who needed advance warning to take part in any research activity [17, 35, 50, 58, 63]. In addition, intervention teams often had to wait and check if the required number of patients had been recruited in each step before proceeding to the next one, leading to cumulative delays. Planning for these additional logistic challenges is therefore critical for teams thinking of embarking on SWT. Researchers should also consider that phased implementation is possible within the context of parallel cluster RCT, and that examples of this are now available [39, 47, 49].

#### Variability of implementation intensity over time

‘The way this was worked out was that there were intervention teams in each district and so they worked with (…) the first step of facilities. They did on-site training and on-site mentoring, and then the frequency of mentoring reduced. And then six months later they had to start the next group of six… And so ultimately, of course, as time passes they have more and more things to do and eventually less and less time for the new facilities coming in. Although they may get better at doing it, because they have more experience…’ (Interview 6)

In lengthy trials of interventions with heavy training or support components, the intensity with which an intervention is delivered may vary over time. For example, the workload of intervention teams may increase as more clusters step into the intervention period [50, 63]. In the BHOMA trial, the quality improvement intervention included an initial training followed by ongoing mentoring using review of primary health centre data, and the intervention team had a heavier workload towards the end [63]. We think that the consequences of such effects are largely trial-specific. In some studies, phased implementation led to increased intensity as teams become better at delivering the intervention; in others, accumulating workload caused intervention fatigue and a decrease in intensity. It may be possible to document this by collecting data on specific features of the intervention (coverage or quality measures, for example) to quantify and model the intervention’s intensity and its relationship with outcomes of interest (although published trials show few examples of this) [4].

#### Changes in intervention models on the basis of experience

‘So the argument was always given as, ‘well, we would have never managed to do everywhere at once, so we randomly picked some villages from where to start, and this will also helps us to better understand if it works or not, and learn about the process along the way to make sure that by the time it comes to your village, the insurance scheme runs better than when we started’. Because, obviously, we also adjusted the scheme as we moved along. Small things in the communication campaign, in the organization and so on’. (Interview 1)

‘In a stepped wedge, you have more opportunity to learn from what may have gone wrong previously. Or in our case we could see ‘run-charts’ of the practices, and at least get an estimate of whether it worked, so there would have been temptation in case it didn’t work, that we would tweak the intervention or try to make it better. So we had a protocol for what we would do in each practice at each point in time to avoid that’. (Interview 2)

‘There has to be a logically acceptable margin of variability for the intervention. (…) If it’s a drug, that’s not a problem. But if people need to be sensitised, trained… well, people don’t all react the same. In our study about falls (in the elderly), towards the end, we had a meeting of all the participating clinics, which was really about sharing experiences… We asked them ‘how did you set up the intervention?’, ‘what happened afterwards?’, ‘what did you create together?’ Members of the clinical teams shared their experiences. That created a kind of dynamic, you see… Each team took ownership of the intervention, but they did this in different ways, and so there will always be the introduction of random variability. And that is extraordinary… That’s the richness of it all… That's what makes it worth it. But of course for ‘methodological purists’ that’s no good at all, it’s not measurable, it introduces variability. But it’s not like ‘I take a drug or I don’t take it’, I receive an intervention, I take it, appropriate it, and I implement it with degrees of variation. (…) The paradox with these kinds of interventions is that they have to be somewhat regulated, but if people do not own them they will never be used later on, for real, and they will never be effective. People need to have that margin of appropriation, of adaptation. If they don’t have it, the battle is lost’. (Interview 5)

Guidelines for the development of complex interventions emphasise the need to define and protocolise activities before their implementation in order to standardise delivery and enhance replicability [11]. However, SWT are often conducted by teams with strong interests in ‘real world’ operational research, which is necessarily inflected by a preference for ‘learning by doing’. Many trials of complex interventions also face unforeseen events because processes are never as controlled in reality as they are in a laboratory [57]. Because SWT involve phased implementation and sometimes build upon routinely collected data, teams may face a tension between protocolising interventions and adapting them as they go along. For example, the DQIP intervention team was not blinded to allocation and could develop a sense of whether it was working from data on the performance of participating surgeries. This made it more tempting to ‘tweak’ the intervention in case it did not work. To remedy this, they put in place a protocol recommending actions at each point in the data collection process [17]. ‘Tweaking’ did take place in the Burkina Faso intervention: the core of the intervention (insurance) did not change over the trial period, but the communication campaign to promote it evolved over time, with the possibility that this may have affected the trial’s outcomes [14]. As a group, we think that it is important to protocolise complex interventions at the beginning, but with the understanding that tweaking or refining - rather than entirely redesigning - might occur; this tweaking can be documented, and, for some interventions, is necessary to ensure local ownership and long-term sustainability.

#### Changes in policy and clinical guidelines

‘We also planned another stepped wedge trial, and we had funding for it, but we had to give the money back because in the meantime there was a competing intervention started by the health board where we wanted to do the trial, and so that meant that we just couldn’t do it. That would also not be great in a two-arm trial, but it may be particularly bad in a stepped wedge trial’. (Interview 2)

Another concern linked with phased implementation is the possibility of policy or clinical guidelines changing during the study timeframe. This has potentially more serious consequences for a SWT than for a parallel CRT: in a parallel CRT, the introduction of a new policy is significant, but would be expected to influence both intervention and control clusters in the same manner. In a SWT, the policy change could change the outcome of the trial dramatically, depending on the proportion of clusters that have crossed over into the intervention phase. For example, the threshold CD4 count for antiretroviral eligibility changed during the study conducted in antenatal clinics in Zambia [35]. In this case, the researchers decided to maintain the previous treatment eligibility cut-off for the evaluation. However, such a decision may not always be possible if the change is mandatory. In some cases the timeframe for completing a stepped wedge trial might also be extended to a duration that compromises the usefulness of its findings: practice may change, guidelines may evolve, or there may be concerns about changing the intervention to reflect new knowledge without vitiating the purpose of the study design.

#### Attrition

Some researchers have suggested that when geographical clusters or health facilities consent to participate in a stepped wedge trial and are randomised to a later intervention start date, they may lose interest and drop out [18]. To prevent this, some intervention teams have actively developed strategies to keep clusters engaged in their trials. For example, the DQIP intervention team gave a one-off financial incentive in line with local research governance regulations to all participating healthcare practices, and also kept them informed of the progress of the study with newsletters [17]. The CONFUCIUS study team organised regular pre-intervention meetings with clusters (surgical wards) in control phases to keep them engaged [50]. Of the 10 stepped wedge trials completed between 2010 and 2014 included in the review conducted for this series, only one lost clusters to follow-up (three of 68 households recruited). This suggests that there is probably no increased risk of attrition with this design [4, 28].

#### Lags in implementation and effect

‘We were trying to see how long it takes after a site ‘steps in’, to when you can consider that the intervention is fully implemented, because that lag time is quite important to calculate’. (Interview 6)

‘What we did for our study on falls, where the intervention was a program of training and reflection within the clinical service, was to use what we called ‘transition time’, that is, we didn’t consider that the entire clinical service was trained after the main referring staff had been trained. We couldn’t assume that… So we told them, ‘you have three months to set things up’, and then we considered that the clinical team was trained up’. (Interview 5)

How does one decide that a cluster - a group of people or a health facility - is fully in receipt of an intervention, and when it can realistically be expected to have ‘worked’? These questions are especially complicated when interventions have multiple components and may take time to be internalised. The issues are common to individually randomised trials, SWT and parallel group CRT [27]. Two typical solutions are to wait until all training components have been delivered, and to allow groups a lag time to settle into the intervention, after which they are considered part of an intervention ‘step’. In common with other CRT, researchers may also factor in a lag in order to allow time for a plausible population-level effect to be observed. Quantifying this time-to-effect can be difficult. The BHOMA team found it challenging to determine how long after their quality of care intervention they might realistically expect an effect of the intervention and an effect on adult mortality. This led them to suggest that stepped wedge designs might be better suited to measuring the effects of interventions with shorter rather than longer lag effects.

The phased implementation implied by SWT requires careful planning: approaching geographic clusters or clinics, collecting data, implementing the intervention, keeping control clusters engaged enough to stay in the trial, and considering the impact of lag times on sample size requirements, analyses, duration, and cost. On balance, both simultaneous and phased implementation pose challenges that need to be appraised on a case-by-case basis, and it is not entirely clear that SWT win in terms of logistic convenience.

### Ethical dimensions

‘Really, the expectation was that whatever we were doing we would be improving things (…) and so they were less keen for us to do it where we had some primary care centres that didn’t get the intervention’. (Interview 6)

‘This thing that all practices were to get the intervention, that was attractive. That was probably the most attractive about it’. (Interview 2)

‘… If I was working on something else or working on the implementation of performance-based financing or a malaria control campaign, then I would still say, ‘that’s really an interesting design, and it makes everybody quite happy because in the end (…) everybody gets the intervention and it’s easier to justify than keeping some people constantly as controls’. (Interview 1)

SWT are subject to the same foundational ethical principles as all clinical research: respect for persons, beneficence, justice, and respect for communities [67]. In addition, a range of specific ethical considerations - including the identification of the trial ‘subject’, the need for informed consent [38], the potential role of cluster gatekeepers, and the protection of vulnerable populations [16, 20, 24, 31, 44, 45, 67] are common to both stepped wedge and parallel CRT.

In this section, we examine ethical questions that are of particular concern to the stepped-wedge CRT. First, we consider whether the evidence in favour of the experimental intervention raises concerns about the ethical permissibility of the SWT design (or concerns about the applicability of our current ethical principles for the assessment of the SWT). Second, we consider whether the idea that providing control subjects with an intervention eventually - that is, delaying its provision to control groups - is a persuasive reason to favour the SWT over other trial designs.

#### Evidence and equipoise

First, the SWT design is based on the idea that an intervention is likely to be effective, and therefore aims to end with the implementation of the intervention. In other words, whether implemented on the basis of new guidelines or on the basis of researchers’ beliefs, the interventions that have been tested in SWT tend to have been accompanied by some conviction that they will do good, and there is a sense that the balance of opinion falls further away from ‘equally distributed uncertainty’ than it does in parallel group trials [1, 3, 7, 10, 15, 22, 25, 26, 33, 34, 41, 48, 51, 62, 64–66]. Implementers used expressions such as ‘we thought it was good, but we didn’t know how good’ (Interview 2), or ‘We felt that it was going to be successful’ (Interview 5).

The balance of evidence in favour of the intervention in a SWT raises an interesting ethical tension. The ethical permissibility of a trial is often thought to depend, at least in part, on the existence of a state of equipoise [32]. That is, equipoise depends on uncertainty or disagreement [3, 10, 22, 25, 34, 51, 65]. But, as the quotations above illustrate, interventions examined using SWT seem likely to be beneficial [13, 25, 34, 62, 65]. This is problematic because if equipoise has already been disturbed, the trial does not seem to meet ethical requirements.

How can we resolve this tension? One possible response is to suggest that equipoise is ill-equipped to assess the permissibility of a SWT. There is some support for this position in the literature. Objections to equipoise include the idea that it has proven hard to delineate and does not quite fit with a public health perspective [1, 7, 26, 41, 48]. Opinions on the potential benefits of an intervention are held by researchers (traditionally labelled as the ‘expert’ group), but also by clinicians, policy-makers, and participants [1, 33, 41, 66]. All four may not agree [66]. Opinions also change with accrual of information and depend not only on the efficacy of the intervention, but also on the trade-off between benefits and harms [41]. Finally, implementation researchers may feel that equipoise is less of an issue for them, and that randomisation is simply a way to ensure *fairness* in allocation, especially in the context of scarce resources, and to assess impact more rigorously.

These responses are not persuasive. Equipoise aims to ensure the appropriate treatment of subjects in all arms of a trial. It also helps to ensure that subjects in the control arm are not unduly deprived of the experimental intervention. Abandoning the requirement of equipoise would not provide research subjects in SWT with these protections, and we think that an ethical principle helping to ensure the appropriate treatment of research subjects is an important part of the ethical assessment of the SWT. More generally, abandoning equipoise would not help to explain when the risks of a stepped wedge trial stand in reasonable relation to the knowledge to be gained.

Perhaps a more constructive solution would be to consider whether the evidence in favour of interventions assessed in an SWT is sufficient to suggest that equipoise has been disturbed at the outset. That is, we might consider the possibility that the intervention under test may still be either ineffective in a particular setting or that it may lead to harm, irrespective of an *a priori* belief in its benefits. Equipoise may apply despite the fact that there is confidence that the intervention under test will ‘work’. In the case of a SWT, the uncertainty might lie in the degree of effect, balance of benefit and harm, cost utility, or effectiveness in a specific operational milieu or at scale. For example, implementation of an intervention might be based on a consensus that it would be beneficial, but there may be uncertainty about its potential effectiveness when rolled out in a given institutional and human resources context. There may also be multiple potential outcomes. Different aspirations for interventional effect are common in public health interventions and are influenced by individual and political perspectives [53].

#### Permissibility of delaying effective treatment

A second ethical question concerns the idea that the stepped wedge design mitigates concerns about the appropriate treatment of research subjects, especially those in the control groups. A perceived advantage of the design is that control groups are certain to receive the intervention eventually (although this is so, it glosses the fact that a given individual in a control group might not actually receive the intervention, which may happen before they join or after they leave). This is thought to address concerns about unjustifiably depriving participants of the intervention being tested [9, 62]. Thirteen of the 31 stepped wedge trial results or protocol articles published between 2010 and 2014 justified their choice of design by invoking the idea that all clusters would eventually receive the intervention, and an *a priori* belief in its benefit [2, 5, 13, 25, 34, 36, 37, 50, 56, 61, 62, 65].

This raises an important ethical question: if it is impermissible to deprive a control group of an intervention, then what, if anything, makes it permissible to delay the intervention to the same control group? Do researchers have an ethical mandate to deprive control groups of an intervention - even for a limited time - in the interests of testing its effectiveness? There is no ethical argument to explain why it should be permissible to deprive participants temporarily of an effective intervention [7]. In the absence of this sort of argument, we think that delayed access is also problematic.

#### Other considerations

In a parallel group trial, control groups are protected from unnecessary roll-out and unpredicted harms. Depending on the design and duration of the trial, a stepped wedge design may lead to a minimum quantum of participants being exposed to the intervention to achieve a parsimonious evaluation. However, in circumstances in which a SWT needs a larger sample size or takes longer than a comparable parallel trial, it will, in fact, expose more people to the control phase than a parallel group trial, which is clearly problematic [10].

Conversely, participants in a control group may be unjustifiably deprived of the benefits of an intervention. If a parallel group trial confirms the efficacy of an intervention, the intervention would generally merit roll-out and the existing control group would usually be the first candidate for introduction. Here ethics blend with logistics: the funding and timescale for a trial do not usually allow for subsequent replication, the trial findings may not be interpreted as a mandate for roll-out, and the implementers of a trial intervention may not be positioned appropriately to enact it on a larger scale [51]. Compared with this lack of assurance, a stepped wedge design can at least guarantee implementation in control groups.

Given our discussion of the background to implementation in terms of equipoise, one might argue, on the other hand, that the stepped wedge design could protect control groups from receipt of an unsuccessful intervention. If the analysis of a SWT involves the use of monitoring data - if it is unblinded and interim analysis is not restricted as it is in the case of a parallel group design - it is conceivable that implementers might stop roll out before control groups have stepped into the intervention. This would presumably be contingent on similar stopping rules to those applied to parallel group trials, but it might in some situations lead to less exposure and, conceivably, less harm if the intervention has negative effects.

Where does this leave us? Assessing the harms and benefits of the stepped wedge design raises similar questions to those arising in parallel CRT and RCT. We think that justifications based on delayed intervention are unpersuasive. We also think that, like other RCT, SWT require equipoise: having some evidence in favour of an intervention does not make the design impermissible, but requires researchers to be particularly explicit about why equipoise still obtains in light of existing evidence.

### Political dimensions

*‘*From our perspective in a National Public Health Centre, when an intervention is rolled out, often there is some evidence, possibly even from the same setting, that shows some degree of effectiveness, so it’s a given that it will be implemented. Or sometimes there is an *a priori* belief and you can’t really challenge that, it will happen anyway. (…) If we believe it works… And I suppose a lot of stepped-wedge designs are informed by that… If you are going to do it everywhere then the choice is either a before-and-after or a stepped wedge design’. (Interview 4)

In a scenario in which a policy decision has been taken to implement an intervention, phased, randomised roll-out provides an opportunity for a more rigorous evaluation than a before-and-after study. This is a powerful argument in favour of the stepped wedge design, but, interestingly, it was only invoked in one of the 31 trials reviewed in this series [19], and one of our case studies [58].

Instead, the most commonly used argument in favour of SWT by researchers themselves is that having a temporary control group is more palatable to participants than completely denying the intervention to the control group:

‘When you do the type of interventions that I do, in clinical service improvement, it is difficult to do a study where you have one arm where nothing is done, and the other arm where something is done. I work in geriatrics, often with hospitals that are not necessarily university hospitals but smaller centres, so telling them ‘the intervention will be implemented later in time, but you will get it’ is a good argument. One day they will have something… Until then they must be patient, but one day they will have something… And that is important for clinicians’. (Interview 5)

‘I think they’ve almost forgotten that it’s a stepped wedge trial, they just think of it as a rolled out evaluation. And I see that a lot… I see a lot of people who talk about stepped wedges… They don’t really mean it as a stepped wedge trial… I have quite a lot of people contacting me and saying ‘I want to do a stepped wedge trial’, but they don’t really want to do that… What they mean is they want to roll out an intervention and evaluate it and somehow they want to do a before-and-after evaluation of a rolled out thing. But they don’t really want to do it as a randomised trial (…)’. (Interview 6)

The use of the stepped wedge design may help to reassure participants and institutions that they will benefit from the intervention and that the phasing will be done fairly, without bias towards particular communities or facilities, increasing the likelihood that they will agree to participate [30, 55]. This point is often offered as an ethical justification for adopting a stepped wedge design, but is in fact logistic (about avoiding attrition) and political (about enhancing the social acceptability of trials).

Anthropologists studying the rise of trials argue that the social, political and economic conditions created by unequal access to health and research resources and decision-making power in high-, middle-, or low-income settings constitute a variable terrain that shifts the relationships between so-called autonomous research subjects, informed consent, and researchers [42]. Ethics and methods are modified to fit the experimental data required to construct the ‘theatre of proof’ of contemporary evaluation through CRT or, for economists, ‘randomised evaluations’ [40, 42, 68]. For the sake of discussion, let us call this ‘trial creep’, always allowing that it may be entirely justifiable from the perspective of evaluators.

An important question is whether the stepped wedge design might exacerbate or mitigate the contentious effects of trial creep. The increasing use of SWT might boost the proliferation of trials more generally, especially in global health research (the thin end of the wedge, if we may be forgiven for saying so). This is not just an academic question about the transferability of methods from clinical research to the evaluation of complex social interventions, about which much has been written [11, 53]. One might argue that the push for randomised evaluations of health and development interventions raises the possibility that areas and clinical services in low- and middle-income settings might become sites for poorly justified experimentation, when resources might be more fairly allocated to simply providing services. External funders and researchers might vest limited decision-making power with local communities and health providers, and a premium on randomised evaluations might delegitimize alternative research strategies, particularly the sort of observational research that local actors might be more comfortable with. Many of the essential questions for global health are operational and must be answered against a backdrop of poverty and deep social inequity. As Farmer argues, flexibility, understanding context, and ensuring local ownership are central to answering them [21].

When conducted on sound ethical principles, both observational research and trials can provide services that otherwise would not exist and improve health. But if we want to improve policy and practice through evaluation, designs such as the stepped wedge which include counterfactuals and also respond to logistic and social concerns should be embraced more widely by the evaluation community. In many contexts they may be an appropriate response to the challenge of making designs address social and clinical realities. To see them as subaltern to parallel trials may be to miss the point, and they might usefully allow more control by local actors and institutions concerned about denying benefits to a control group.

Our study has strengths and limitations. It provides an account of the logistic benefits and drawbacks of SWT that is grounded in researchers’ own experiences. It also offers a discussion of the ethics of SWT informed by the broader literature on trial ethics. The purposive selection of case studies is a potential limitation. Clearly, the views of this group of authors may not represent the experiences of all researchers. Our case studies were also limited to the field of public health and represent only a certain range of SWT designs. The nature and degree of evidence required to disturb equipoise in this context may differ from others.

## Conclusions

Our article proposed three main arguments in relation to the logistics, ethics, and politics of SWT in the real world. First, the phased implementation of interventions may alleviate problems linked to simultaneous roll-out, but also brings new challenges, particularly those linked to sequential intervention activities, estimating lag times in implementation and effect, and dealing with changes in policy during the trial period. Second, SWT do not release investigators from the duty of equipoise; without it, randomising participants to a control condition, even for a short period of time, remains ethically problematic. For SWT, equipoise is likely to lie in the degree of effect, balance of benefit and harm, cost utility, or effectiveness in a specific operational environment. The third, related point is that the strongest arguments for a stepped wedge design are logistic and political rather than ethical. The design is advantageous when simultaneous roll-out is infeasible or impractical, and when it increases the acceptability of using counterfactuals in domains in which this is uncommon.

## Abbreviations

BHOMA: Better Health Outcomes through Mentoring and Assessment
CRT: cluster randomised controlled trial
DQIP: Data-driven Quality Improvement in Primary Care
GP: general practice
HIV: Human Immunodeficiency Virus
SWT: stepped wedge cluster randomised controlled trial
UK: United Kingdom
